# Bluetongue Virus in France: An Illustration of the European and Mediterranean Context since the 2000s

**DOI:** 10.3390/v11070672

**Published:** 2019-07-23

**Authors:** Cindy Kundlacz, Grégory Caignard, Corinne Sailleau, Cyril Viarouge, Lydie Postic, Damien Vitour, Stéphan Zientara, Emmanuel Breard

**Affiliations:** UMR Virologie, INRA, Ecole Nationale Vétérinaire d’Alfort, laboratoire de santé animale d’Alfort, ANSES, Université Paris-Est, 94700 Maisons-Alfort, France

**Keywords:** Bluetongue virus, Europe, Mediterranean Basin, France, control measures, 20 years

## Abstract

Bluetongue (BT) is a non-contagious animal disease transmitted by midges of the *Culicoides* genus. The etiological agent is the BT virus (BTV) that induces a variety of clinical signs in wild or domestic ruminants. BT is included in the notifiable diseases list of the World Organization for Animal Health (OIE) due to its health impact on domestic ruminants. A total of 27 BTV serotypes have been described and additional serotypes have recently been identified. Since the 2000s, the distribution of BTV has changed in Europe and in the Mediterranean Basin, with continuous BTV incursions involving various BTV serotypes and strains. These BTV strains, depending on their origin, have emerged and spread through various routes in the Mediterranean Basin and/or in Europe. Consequently, control measures have been put in place in France to eradicate the virus or circumscribe its spread. These measures mainly consist of assessing virus movements and the vaccination of domestic ruminants. Many vaccination campaigns were first carried out in Europe using attenuated vaccines and, in a second period, using exclusively inactivated vaccines. This review focuses on the history of the various BTV strain incursions in France since the 2000s, describing strain characteristics, their origins, and the different routes of spread in Europe and/or in the Mediterranean Basin. The control measures implemented to address this disease are also discussed. Finally, we explain the circumstances leading to the change in the BTV status of France from BTV-free in 2000 to an enzootic status since 2018.

## 1. Introduction 

Bluetongue virus (BTV) belongs to the *Orbivirus* genus within the *Reoviridae* family. This virus is the etiological agent of Bluetongue (BT), a non-contagious disease transmitted by blood-feeding midges of the *Culicoides* genus [1]. *Culicoides imicola* is known to be the main BTV vector in Africa and in the Mediterranean Basin [2]. BTV infects a wide range of wild and domestic ruminants and causes a variety of clinical signs. In the most severe cases, BTV can cause hemorrhagic fever and mortality in sheep, which are the most BTV-sensitive ruminant species. BTV can also induce fever, depression, respiratory distress, and anorexia [1,3,4]. Although goats and cattle are susceptible to BTV infection, they generally do not show signs of disease, except for outbreaks that occur in non-endemic areas [5] where mild or subclinical signs have been reported for both species. Cattle show longer periods of BTV viremia than sheep and are considered to be reservoirs of the virus [1,6,7].

BTV is a double-capsid virus with a genome of 10 double-stranded RNA segments encoding seven structural (VP1 to VP7) and five non-structural proteins (NS1 to NS5) [8,9]. The recognition of BTV serotypes is mainly based on specific interactions of neutralizing antibodies (NAs) with VP2 proteins that form the outer capsid [10,11]. VP2 is the most variable BTV protein and is considered to be the major serotype-defining protein of the virus [12]. In total, 27 serotypes of BTV have been described [13], and additional novel serotypes have recently been identified, particularly in countries bordering the Mediterranean Basin [4,14,15,16,17,18]. Within the same serotype, two major geographic groups of BTVs have been designated as eastern or western topotypes. The eastern strains include viruses from Australia and the Middle/Far East, whereas the western viruses originate from Africa and the Americas [19]. Eastern and western viruses have both been involved in the different European or Mediterranean BTV incursions (particularly in France), through various routes. 

Until 1998, BTV was restricted to between 40° and 50° North latitude and between 20° and 30° South latitude, the latter being confined to the tropical and subtropical regions and coinciding with certain species of the *Culicoides* vectors of the disease [20]. In Europe, BTV outbreaks were sporadically observed in the Mediterranean Basin (40° North latitude). Since the 2000s, this global distribution of BTV has changed, with continuous emergence particularly in Europe and in the Mediterranean Basin. These BTV incursions have involved different serotypes: BTV-1, 2, 3, 4, 6, 8, 9, 11, and 16. As a consequence, France has shifted from a BTV-free status in 2000 to an enzootic status for serotypes 4 and 8 since January 2018.

BT has been included in the notifiable diseases list of the World Organization for Animal Health (OIE) since the 1960s due to its health impact on domestic ruminants. Importantly, outbreaks caused by non-virulent (atypical) strains (BTV-25, 26, 27 and new, recently identified BTV strains that could also be prototypes of new serotypes [4,14,15,16,17,18]) do not require notification to the OIE, nor restrictions to animal trade. For other epizootic events in Europe, involving the 24 classical serotypes, control measures can be implemented in order to eradicate the virus or circumscribe its spread. These measures mainly consist in herd vaccination and control of animal movements. Vaccination is the most efficient veterinary method against BTV and is considered to be the best option to: (1) reduce clinical disease and losses, (2) control the spread of the disease and protect virus-free territories and neighboring countries, and (3) to facilitate safe trade in live animals.

This article describes BTV incursions into France and the control measures set up over the last two decades to address this animal disease. It reports on the various origins and ways the virus has been introduced and describes the epidemiological characteristics of the various BTV strains involved in BT outbreaks in France. The history of BTV reveals how a European country like France has shifted, within 20 years, from a BTV-free status to an enzootic status.

## 2. First Period: Bluetongue Virus (BTV) Incursions in Countries Bordering the Mediterranean Basin 

**Figure 1:**
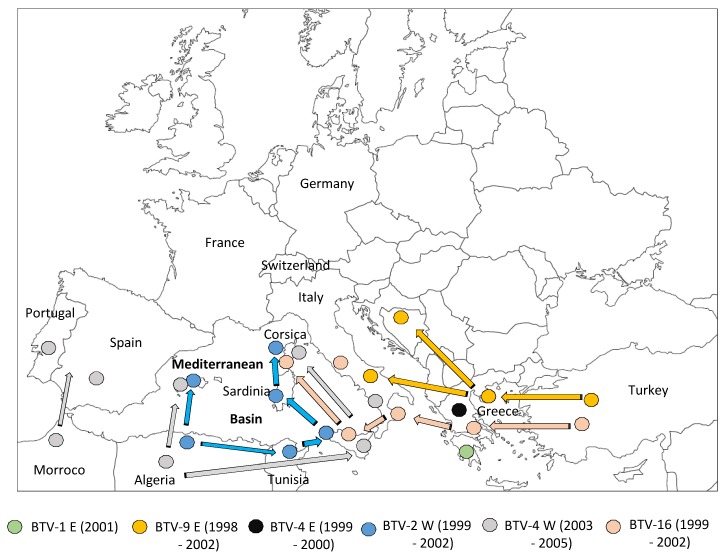
Bluetongue virus (BTV) context from 1998 to 2005. Spread of BTV serotypes and strains (BTV-x) during the period indicated in brackets. E: Eastern strain; W: Western strain.

Until 1998, incursions of BTV into the Mediterranean Basin or South Europe were relatively infrequent and did not persist. From 1998 to 2005, at least seven strains from five different BTV serotypes (serotypes 1, 2, 4, 9, and 16) were detected in the Mediterranean Basin [21] (Figure 1). This new distribution of strains and serotypes is due to various factors, including increased global trade and climate change [1,22,23]. Climate change may be involved in this new pattern of BTV distribution by its impact on the vectorial capacity of resident *Culicoides* insect populations in previously virus-free regions, such as much of the Mediterranean Basin and European countries [1]. These BTV strains have been introduced to the Mediterranean Basin and to Europe through two corridors: eastern viruses via Turkey and Greece, and western viruses via North Africa [24]. In the case of western viruses, BTV first spread to North Africa, then to the Mediterranean islands, and finally to southern European countries.

The BTV situation has changed since 1998. A BTV-9 (eastern strain) incursion was first reported in sheep in the Greek islands from which it spread to European Turkey, Balkan countries, and Italy in 2000–2002 [25]. BTV-9 persisted in these regions for five years and highlighted a route of BTV introduction into Mediterranean countries, as well as the fact that BTV was capable of overwintering in European countries. During the same period, different BTV serotypes were reported in Mediterranean countries: BTV-4 and 16 in Greece (1999–2000), BTV-16 in Italy (2002), and BTV-1 on a Greek Island (Lesbos) in 2001 [26,27]. However, none of these eastern BTV strains persisted or spread to other areas. Nonetheless, a BTV-2 strain, originating from sub-Saharan Africa, circulated in North Africa (Tunisia (1999 and 2000)) before being reported in the Balearic Islands, Sicily, Sardinia, (2000), and finally in Corsica in 2000 [28]. Altogether, these BTV-2 epizootics have revealed another corridor (from South to North) for BTV emergence in the Mediterranean Basin.

In response to the BTV-2 incursion in 2000 in Corsica (49 sheep herds infected), the French Veterinary Authorities set up two vaccination campaigns, which were both carried out from December to April in 2000–2001 and 2001–2002, using a commercial South African attenuated vaccine. The first vaccination campaign did not provide full protection for all sheep since 335 sheep herds were found to be infected with BTV-2 (a 7-fold increase compared to 2000) during summer and autumn of 2001 in Corsica. This demonstrated that BTV-2 had persisted during the winter and re-emerged the year after. However, no cases of BTV-2 were observed in 2002 in Corsica, indicating that the two vaccination campaigns were successful in protecting sheep from infection [29].

In 2003, the same course of events as that observed with BTV-2 (i.e., BTV spread from North Africa to the South of Europe) occurred with a BTV-4 strain, detected first in Sardinia, and then in Corsica and Minorca. In 2004, this BTV-4 strain was also isolated in Morocco (confirming the African origin of the strain) and later in Spain and Portugal, showing that BTV can spread northward via the west of the Mediterranean Basin, from North Africa into Spain. BTV-4 was also detected in Greece in 1999 and 2000, suggesting another route of incursion of this serotype into Europe. Analysis of genome nucleotide sequences from the different BTV-4 field strains isolated in the Mediterranean countries showed that the BTV-4 isolates from Corsica, Morocco, Spain, and Italy (2003–2004) were distantly related to the Greek strains isolated in 1979, 1999, and 2000, confirming the spread of two lineages of BTV-4 in the Mediterranean Basin having eastern or southern origins [30]. In Corsica, two vaccination campaigns with an attenuated vaccine carried out in 2003 and 2004 eradicated this strain in the Island. 

In 2004, BTV-16 was isolated from sheep in Corsica. The genome sequence analysis showed that this BTV-16 was identical to the BTV-16 attenuated strain present in a vaccine batch that had been produced in Onderstepoort (South Africa). At the same time, this attenuated vaccine strain was used in Italy and unvaccinated animals became ill as a result of BTV infection [31]. During 2002–2004, BTV-16 strains were also detected and isolated from the field in Turkey, Sicily, and mainland Italy. Phylogenic analyses demonstrated that all these field BTV-16 strains derived from the attenuated vaccine produced in South Africa, from a strain originally isolated in Pakistan [32]. Attenuated vaccines are live viruses with a reduced capacity to grow in vivo and, consequently, to cause disease. However, high viremic titers and clinical signs were observed in animals that had been vaccinated with a BTV-16 attenuated strain in Italy. A few months later, this BTV-16 vaccine strain was isolated from animals in areas of Italy previously free of this serotype and finally spread to Corsica, illustrating the fact that attenuated vaccines could be naturally transmitted by vector midges [33]. Fortunately, in Corsica, the BTV-16 strain was no longer detected after 2004 without any prophylactic action, suggesting that this attenuated BTV-16 strain, although able to be transmitted by *Culicoides*, lost its original properties to efficiently spread in the field. Following these observations, after the use of the attenuated BTV-16 vaccine, European Union countries decided to use only inactivated vaccines moving forward. 

In 2007, another BTV serotype spread northward, from Africa to Europe. A strain of BTV-1 present in North Africa in 2006 (detected in 2006 in Morocco and Algeria) spread to Sardinia (as already observed for BTV-2 and 4). However, this BTV-1 strain also spread northward via the west of the Mediterranean Basin (Gibraltar in 2007) to Spain and Portugal, to finally be detected in the South of France [23]. To date, more than 6,000 BTV-1-affected farms have been identified in France. Moreover, the simultaneous presence of BTV-1 and BTV-8 in southwestern France in 2008 (see next section) also led to co-infections and, as a consequence, to re-assortment events between BTV-1 and BTV-8 strains (Figure 3) [34]. An inactivated vaccine for BTV-1 became commercially available in 2008 and was used in mainland France. The last case of BTV-1 in continental France was identified in June 2010. After this date, BTV-1 was not isolated from the field. The BTV-8 and BTV-1 epizootics in France had a considerable impact on livestock and led to economic losses as well as significant costs due to the control measures put in place (movement control and mass vaccination of domestic ruminants) [35]. Following mandatory vaccination campaigns, both BTV-1 and BTV-8 were eradicated in 2010 in France, and the country regained a BTV-free status in 2012 [36].

In 2012, a novel strain of BTV-4 was identified in Sardinia. This strain has been shown to be closely related to BTV-4 isolated in Tunisia in 2007 and 2009 [37]. All these BTV-4 strains were western strains and differ from the homolog strains responsible for the 2003–2005 incursions in Mediterranean countries, and from the BTV-4 strain detected in Greece in 1999. These novel BTV-4 strains constitute a third group of strains identified in this area because they shared high_sequence homology (segment 5; 99% nucleotide homology) with the BTV-1 strains circulating at the same time in the Mediterranean Basin. The BTV-4 reassortant did not spread out of Sardinia (Figure 3). Only wild-type BTV-1 spread northward to Corsica in 2013 [38]. Once again, genome sequencing demonstrated that this BTV-1 strain spread from Tunisia (detected in 2011) through Sardinia and had the same origin as BTV-1 present in the Maghreb in 2006 and detected in France in 2007 [24]. After the vaccination of domestic ruminants during winter 2013–2014, this serotype was no longer detected in Corsica after 2014. It has not crossed the Mediterranean Sea to mainland France, as already observed for the BTV-2 and BTV-4 wild-type strains present in Corsica in the 2000s. All these strains originating from Africa are able to spread periodically to southern European countries but ultimately did not persist nor spread to northern European areas. 

Since 2016, a virulent form of BTV-3 detected in sheep showing clinical signs has spread in Tunisia and, following the scenario already observed, this strain was then detected in Sicily and in the South of Sardinia in 2018 [39]. Given the last 20 years of BTV history in the Mediterranean Basin, we presume that the risk that this serotype strain will appear in the near future in Corsica or further north is very high.

## 3. The Second Period: A New Area of Emergence in Northern Europe Changed the BTV Introduction Rules in 2006 

Concomitantly with these introductions of BTV from countries bordering the Mediterranean Basin, European countries faced new BTV incursions of unknown origin.

**Figure 2:**
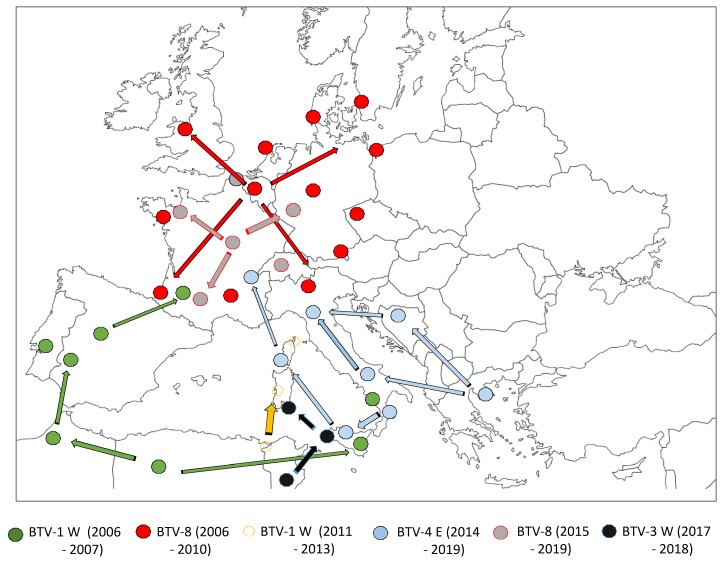
BTV spread from 2006 to 2019. Spread of BTV serotypes and strains (BTV-x) during the period indicated in brackets. E: Eastern strain; W: Western strain.

In 2006, a new BTV serotype emerging and spreading in northern Europe changed views concerning the BTV introduction pathway into Europe. The first cases of BTV-8 were identified in the Netherlands where 456 outbreaks were finally reported during 2006. The virus spread to Belgium (695 outbreaks), Germany (885 outbreaks), Northern France (6 outbreaks), and Luxembourg (5 outbreaks) [23] (Figure 2), illustrating that a BTV could emerge and spread in Europe at latitudes greater than 50° N.

The BTV-8 strain during the epizootic in Northern Europe was virulent in sheep but also unexpectedly in cattle [40,41,42,43,44]. Moreover, BTV-8 also had the ability to infect wild ruminant species [45,46]. The disease state associated with this viral strain was also unusual. BTV-8 causes various reproductive disorders such as transient infertility, abortions, and increased farrowing intervals, but also stunted growth, and an alteration in wool quality [40,41]. In addition, this strain could be transmitted from pregnant females to their fetuses through the transplacental route. In infected calves, the clinical signs included blindness, ataxia, inactivity, behavioral abnormalities (dummy calf syndrome), and hydranencephaly [47,48,49]. Spontaneous occurrence of similar abnormalities has also previously been described in cattle in the United States and South Africa, where attenuated virus vaccines have been used [50]. Moreover, studies with Australian BTV strains in pregnant sheep found that BTV crossed the placenta to induce teratogenesis only after strain adaption to cell culture [51]. The origin of this BTV-8 strain is still undetermined as well as the introduction mode into Europe [52,53]. Sequence genome alignments revealed close similarity to a BTV-8 strain isolated in sub-Saharan Africa [19], suggesting African origin. The transplacental transmission properties of this strain may come from a modified live vaccine at that time used in several countries (outside Europe). Following mandatory vaccination campaigns using inactivated vaccines, some European countries regained their BTV-8-free status from 2010 to 2012 (2012 for France).

The spread of BTV-8 in northern Europe could not be linked to *Culicoides imicola*, which is absent from the first affected areas. However, *C. obsoletus, dewulfi, scoticus,* and *chiopterus* autochthonous midges, have been identified as the vectors of BTV-8 in northern and western Europe [54,55]. In contrast to what we would have expected, the winter season did not eradicate the disease. In fact, the virus re-emerged in 2007 simultaneously with the resumption of vector activity, in all affected countries in 2006, from which it rapidly spread to new countries (Denmark, Switzerland, Czech Republic, and the United Kingdom), affecting nearly 60,000 holdings [23]. The 2008 BTV-8 epizootic in Northern Europe is believed to have caused greater economic damage than any previous single serotype BT outbreak [56,57]. In 2008, a major vaccination campaign began in the infected areas of all the European countries concerned, leading to a significant reduction in the number of outbreaks, and thus contained the spread. However, due to high demand in Europe, there were insufficient vaccine doses to protect all the regions against BTV-8. For instance, in France, an increased number of BTV-8 cases was observed in 2008 (24,000 infected holdings) [23]. An additional year of mass vaccination (2009) was necessary to observe a drastic reduction of BTV-8 cases in France (83 outbreaks).

**Figure 3:**
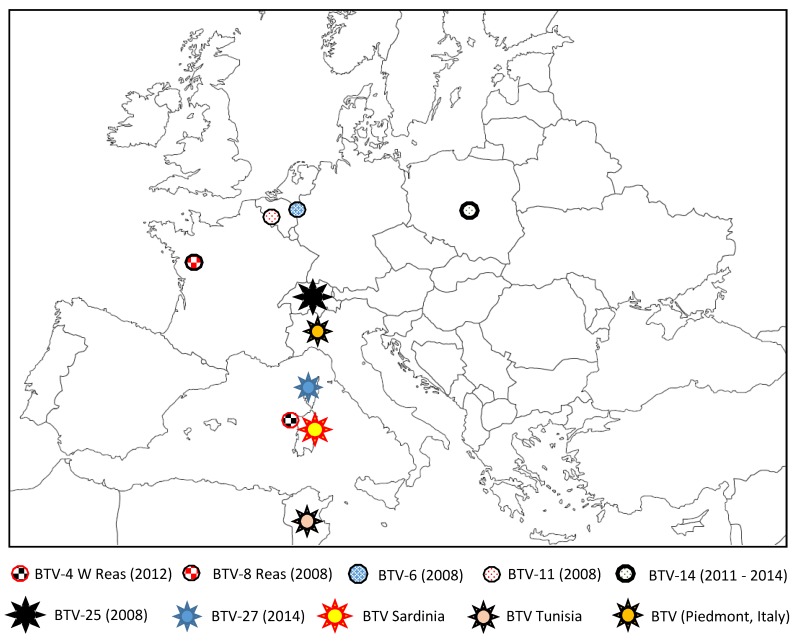
BTV reassortant strains (BTV-4 W Reas and BTV-8 Reas), modified strains (BTV-6, 11 and 14), and novel BTV strains or serotypes detected in Europe (BTV-25, Piedmont) and in the Mediterranean Basin (Sardinia, Corsica, and Tunisia) from 2008 to 2018.

Subsequently, BTV-6, BTV-11, and BTV-14 strains (Figure 3) genetically similar to live modified vaccine strains have been found in northern Europe [58,59,60]. BTV-6 and -11 emerged in 2008, in the same area where BTV-8 emerged in 2006 (in the Netherlands and Belgium). Fortunately, only very limited spread of BTV-6 and -11 were reported in the field in 2008, without clinical signs and, as observed with BTV-16 in Corsica, both BTV strains have no longer been detected. BTV-14 was detected for the first time in 2011 in Russia and in 2012 in Poland [59]. This serotype was present in Poland until 2014, and then no further cases were reported. The introduction origins of these vaccine strains are still unknown.

Three years after the eradication of BTV-8 in France, this serotype was again detected in September 2015 on a cattle and sheep farm in the Allier department [61]. Clinical signs suggestive of BTV (increased temperature, respiratory problems, and facial edema) were observed in a five-year-old ram. Complete sequencing of the viral genome allowed us to determine that it was 99.9% identical to the BTV-8 strain that had circulated in France between 2006 and 2010, suggesting the re-emergence of this strain [62]. The origin of this re-emergence is likely very low-level circulation of this serotype since its first emergence in 2006 [63]. Because there were insufficient doses of inactivated vaccines available in 2015, the French Veterinary Authorities decided to establish control measures to limit the spread of BTV-8 from infected to non-infected areas. Restrictions on livestock movements were implemented and periods without *Culicoides* vector activity were determined. The vector-free period is one criterion to enable safe movements of susceptible livestock. In accordance with the EU regulation (defined in Annexe V of Commission Regulation (EC) No. 1266/2007), vector-free periods are times when it is expected that there is an extremely low risk of BTV transmission. In France, a threshold of catching fewer than five parous female *Culicoides* per trap was set as a limit for determining no adult *Culicoides* activity, and then to define the start and end of vector-free periods.

However, due to the spread of BTV-8 by vectors from infected livestock to neighboring and non-protected ruminants, BTV-8 has rapidly spread in mainland France. Since 2015, nearly 3,000 cases of BTV-8 have been reported in the country. These BTV-8 cases were mainly reported from cattle that have been tested before exportation (personal data), the number of cases is then widely underestimated. Very few clinical signs were observed when compared to the first BTV-8 outbreaks in 2006–2008 in France. This could be explained by the fact that the animals developed partial immunity during the primary infection of 2006, or by the mandatory vaccination campaign put in place until 2010. However, a change in the virulence of the virus cannot be ruled out [64]. By April 2019, BTV-8 had spread northward and eastward and is now present in western Germany, Switzerland, and Belgium. Inactivated vaccines are available, and farmers can vaccinate on a voluntary basis, except for trade where vaccination is compulsory.

## 4. First Eastern BTV Strain Introduction in Mainland France

In November 2016, sheep located in Corsica exhibited clinical signs suggestive of BTV disease. Laboratory analyses allowed us to identify a BTV-4 strain [65]. Sequence analysis showed a close relationship between this strain and another BTV-4 strain isolated in Hungary in 2014 [66], which had been responsible of a large BT outbreak in Greece, the Balkan countries, and Italy (Sicily and Sardinia). Comparison of genome sequences between the BTV-4 strains that circulated in Corsica previously in 2003 and 2016, showed that these two strains are genetically distinct, thus suggesting that in the first report in Corsica of a BTV strain, the virus originated from eastern Europe (Figure 2).

In June 2017, BTV-4 spread across Corsica causing serious illness in unvaccinated sheep. Cattle were also infected but did not show any clinical signs. In November 2017, BTV-4 was detected in the Haute-Savoie department in mainland France [67], from an asymptomatic 15-day-old calf tested before export to Spain. A detection survey was set up with a radius of 10 kilometers from where the first case of BTV-4 had been detected and led to the identification of a second neighboring farm infected by BTV-4. The French authorities established a 150 km restriction zone around the farms, with limits on livestock movements. In addition, an emergency vaccination campaign was implemented in this restriction zone using commercially available inactivated vaccines. Full genome sequencing of this strain showed its close link with the BTV-4 strain that spread in Corsica and Italy during the same year. The origin of the BTV-4 introduction into mainland France was probably Corsica, following cattle imports (during the BTV-4 epizootic in 2017) by the second set of farms found to be BTV-4-infected in Haute-Savoie [67].

Indeed, after having implemented measures aimed at eradication, further BTV-4 outbreaks were reported in other French departments. In this context, vaccination over a large part of France to successfully carry out eradication of this serotype was not possible due to availability of vaccine doses (tens of millions needed), and the cost. Since 2018, BTV-4 has been considered enzootic in France. Like for the BTV-8 enzootic, inactivated vaccines are available and are mainly used for trade.

## 5. Discovery of New BTV Serotypes 

The year 2008 was not only characterized by the occurrence of BTV-6 and -11. In Toggenburg (Switzerland), clinically healthy goats were identified as BTV-positive by RT-PCR. Phylogenetic analyses supported the classification of this BTV serotype as a novel serotype, representing serotype 25 [18]. Novel serotypes have since been identified, particularly in countries bordering the Mediterranean Basin (Sardinia, Piedmont (northwest Italy), and Tunisia) (Figure 3) [4,14,15,16,17,18]. In 2014, a new serotype, BTV-27, was found in goats in Corsica [13]. A prevalence survey distinguished three distinct variants within this new serotype (v01–03), but none of them cause clinical signs [68]. In the field, the v01 variant was more commonly found in comparison with the two others. From 2016 to 2018, BTV-27 was sporadically detected and isolated from asymptomatic goats in Corsica, where only v01 was detected. From cumulative field data from Corsica collected since 2014, cattle as well as sheep are not susceptible to infection with any BTV-27 variant [69].

At the experimental level, the three BTV-27 variants can replicate in mammalian cells (clone of BHK 21 cell line) but not in *Culicoides* cells (KC cells) [69]. Experimental goat infections have revealed that the v02 variant could be transmitted by direct contact between goats, like BTV-26 [70]. This would suggest that the *Culicoides* vector is not necessary for the transmission of this serotype. The observed phenotypes seem to reveal that the three variants of BTV-27 have characteristics closely related to serotypes 25 and 26 [68].

The atypical biological characteristics of this BTV-27 serotype (i.e., cattle and sheep not susceptible for BTV-27 infection, contact transmission between goats, no replication in KC cells, delayed detection, and moderate increase of BTV-RNA and antibodies in BTV-27 infected goats [69]) illustrated those reported for other new serotypes found in Europe and in the Mediterranean Basin (Switzerland, Italy, and Tunisia), and also serotypes detected in Kuwait and China [15,70]. Altogether, these strains constitute a new class of BTV serotypes, which have only been found in asymptomatic small ruminants. Consequently, outbreaks caused by these non-virulent BTV strains do not cause any restrictions to animal trade.

## 6. BTV Vectors

Many factors contribute to the spread of vector-borne diseases, including the presence of infected hosts, competent vectors, and suitable environmental temperatures for the vector to replicate and transmit the pathogen. The vector should also be significantly abundant. Studies performed in the Mediterranean Basin showed that in Sardinia, *Culicoides* fauna comprises the confirmed or probable BTV vectors, *C. Imicola*, *C. Obsoletus*, and *C. Scoticus* (belonging to the subgenus *Avaritia*), *C. Pulicaris*, *C. Punctatus*, and two species of the *Newsteadi* complex [71,72]. In this island, BTV-2 was found in 2001 in *Newsteadi* species between December to May and in *C. Imicola* between September to November in conjunction with the BT epidemic [71,73]. The winter–spring activity period makes the *newsteadi* species the potential candidates for virus overwintering in Sardinia when *C. Imicola* seems to be the vector responsible of the BTV-2 outbreaks. A study, conducted in several Italian regions during BTV-1 and BTV-4 outbreaks in 2012–2014 [73], confirmed BTV positivity for these *Culicoides* species found in Sardinia. Remarkably, high virus genome loads were detected in pools of *newsteadi* from southern Italy, which substantiates the potential role of this species in transmitting BTV-1 and -4 during outbreaks in continental Italy. We might also formulate the hypothesis that the transmission of BTV in certain areas is dependent on the BTV strain introduced: only the BTV-8 strain was capable of overwintering in northern Europe (without *C. Imicola* species) when BTV-6 and -11, that emerged in the same area in 2008 were able to persist. Based on this information, it is clear that different vectors species potentially contribute to BTV transmission according to the ecosystems and the strain involved.

## 7. Conclusion

The epidemiology of BTV in France over the last two decades perfectly illustrates BTV movement patterns in the Mediterranean Basin, and more widely in Europe since the 2000s.

After the rare and sporadic BTV incursions observed in this area before 1998, the situation has worsened with the spread of different BTV strains (mainly from Africa) and their persistence for several years. This also demonstrates that these different strains were able to overwinter in countries bordering the Mediterranean Basin. In areas where at least two BTV strains circulated in the same period (France and Italy), re-assortment events have also been reported. The emergence and spread of BTV-8 in northern Europe from 2006 to 2008 showed that BTV can emerge, spread, and persist in territories known historically as free from this pathogen, with new autochthonous midge species as vectors that had never been in contact with the viruses. Moreover, the teratogenic properties of the BTV-8 strain suggest that this strain could have a modified live vaccine as an origin. The route and the method of introduction of BTV-8 into northern Europe are still unknown. However, since two BTV serotypes that originated from live modified vaccines, BTV-6 and BTV-11, were also detected in 2008 in the same area, this European region is now considered a major pathway of BTV introduction into Europe [74,75]. Fortunately, the majority of these modified BTV strains (BTV-16, -6, -11, and -14) seemed not to be able to persist in the field.

Until 2015, the French authorities took measures to successfully eradicate BTV-1, -2, -4, and -8 using mass vaccination campaigns. Eradication was generally achieved after two or three mass vaccination campaigns. In France, where measures against each BTV incursion were implemented from 2000 to 2013, the control costs were higher and higher each time, and reached a record with the mass vaccination campaigns against BTV serotypes 8 (and 1) carried out in mainland France [57]. However, with the combination of the BTV-8 re-emergence in 2015 and the emergence of BTV-4 in 2017, the French Veterinary Authorities finally decided that both BTV-8 and BTV-4 are considered enzootic in France (since January 2018). One of the direct consequences was the spread of BTV-8 in 2018–2019 to neighboring countries (Germany, Switzerland, and Belgium). It seems that Europe, and particularly European countries bordering the Mediterranean Basin, must now live with BTV.
